# Integrating mHealth in Oncology: Experience in the Province of Trento

**DOI:** 10.2196/jmir.3743

**Published:** 2015-05-13

**Authors:** Enzo Galligioni, Enrico Maria Piras, Michele Galvagni, Claudio Eccher, Silvia Caramatti, Daniela Zanolli, Jonni Santi, Flavio Berloffa, Marco Dianti, Francesca Maines, Mirella Sannicolò, Marco Sandri, Lara Bragantini, Antonella Ferro, Stefano Forti

**Affiliations:** ^1^Medical Oncology Department, Azienda Provinciale per i Servizi SanitariTrentoItaly; ^2^e-Health Research Unit, Fondazione Bruno KesslerTrentoItaly; ^3^Mtt-pro srlRoveretoItaly; ^4^Pharmacy Unit, Azienda Provinciale per i Servizi SanitariTrentoItaly

**Keywords:** health informatics, mobile health, home monitoring, supportive care, patient safety, safe chemotherapy

## Abstract

**Background:**

The potential benefits of the introduction of electronic and mobile health (mHealth) information technologies, to support the safe delivery of intravenous chemotherapy or oral anticancer therapies, could be exponential in the context of a highly integrated computerized system.

**Objective:**

Here we describe a safe therapy mobile (STM) system for the safe delivery of intravenous chemotherapy, and a home monitoring system for monitoring and managing toxicity and improving adherence in patients receiving oral anticancer therapies at home.

**Methods:**

The STM system is fully integrated with the electronic oncological patient record. After the prescription of chemotherapy, specific barcodes are automatically associated with the patient and each drug, and a bedside barcode reader checks the patient, nurse, infusion bag, and drug sequence in order to trace the entire administration process, which is then entered in the patient’s record. The usability and acceptability of the system was investigated by means of a modified questionnaire administered to nurses. The home monitoring system consists of a mobile phone or tablet diary app, which allows patients to record their state of health, the medications taken, their side effects, and a Web dashboard that allows health professionals to check the patient data and monitor toxicity and treatment adherence. A built-in rule-based alarm module notifies health care professionals of critical conditions. Initially developed for chronic patients, the system has been subsequently customized in order to monitor home treatments with capecitabine or sunitinib in cancer patients (Onco-TreC).

**Results:**

The STM system never failed to match the patient/nurse/drug sequence association correctly, and proved to be accurate and reliable in tracing and recording the entire administration process. The questionnaires revealed that the users were generally satisfied and had a positive perception of the system’s usefulness and ease of use, and the quality of their working lives. The pilot studies with the home monitoring system with 43 chronic patients have shown that the approach is reliable and useful for clinicians and patients, but it is also necessary to pay attention to the expectations that mHealth solutions may raise in users. The Onco-TreC version has been successfully laboratory tested, and is now ready for validation.

**Conclusions:**

The STM and Onco-TreC systems are fully integrated with our complex and composite information system, which guarantees privacy, security, interoperability, and real-time communications between patients and health professionals. They need to be validated in order to confirm their positive contribution to the safer administration of anticancer drugs.

## Introduction

Delivering effective and safe treatment is one of the main challenges facing health professionals, and this is particularly important in medical oncology because chemotherapy and target therapies are generally characterized by complex regimens, a high degree of toxicity rates, and a narrow therapeutic window [1]. The process of prescribing, preparing, and administering current chemotherapy treatments is complex, and prescription and administration errors are still common: 7% in the case of adult chemotherapy to 19% in a pediatric setting, and fewer than 2% of these errors are intercepted at the patient bedside [2-4].

The development of new technologies, a safety culture, and evolving workflows have been widely reported, and have been shown to have the potential for reducing medication errors in various health care settings [5]. The information technologies (ITs) introduced over the past 20 years have facilitated patient management, improved the safety and precision of administering cancer treatments safer, and increased the efficiency of the process of ordering, preparing, and administering antineoplastic drugs [5,6]. The use of electronic patient records (EPRs), clinical decision support systems (CDSS), computerized prescriber order entry (CPOE), barcode-assisted medication administration (BCMA) systems, intravenous infusion safety systems (smart pumps), electronic medication administration records (eMARs), and telepharmacy have all been extensively described [5-8] and, although evidence supporting their use in preventing medication errors is limited (particularly in oncology), their potential benefits could become exponential if incorporated in an integrated technological system [5]. This has been highlighted by the Institute of Medicine (IOM), which has recently defined electronic medical record (EMR) systems in which clinical information, decision support tools, and CPOE are closely integrated: “a vital piece of the health information system needed to improve cancer care” [9].

CPOE is the only technology that has been demonstrated to contribute to reducing medication errors in oncology [8,10-14], and is therefore being increasingly used in the case of anti-neoplastic drugs. This has prompted the American Society of Health-System Pharmacists (ASHP) to publish guidelines concerning its use, particularly when integrated with a pharmacy information system [15]. Although it has been reported that CPOE may sometimes lead to increased errors (most of which consist of the wrong cycle number or stage, or wrong height or weight), these can be easily prevented by optimally designed CPOEs integrated in EMR systems, which significantly improve the quality, safety, and efficiency of the complex medication of cancer patients [8,11,13,14,16].

BCMA is the second most frequently implemented technology and is intended to reduce medication errors at a patient’s bedside [17-19]. Its value has been proved in a broad range of patients and numerous organizations including the Food and Drug Administration (FDA), IOM, and ASHP have urged its adoption, although there is a lack of concrete supporting it in anticancer therapy [5,6,18-20]. However, its integration with other systems, such as EMR, CPOE, and eMAR, which can also track appropriate medication use, has been found to be effective in many areas [5,6,21,22] including oncology [7]. Moreover, if CPOE is integrated with a pharmacy information system, BCMA and eMAR are both automatically updated whenever new medication orders are entered or existing orders are modified [6,21].

New developments in cancer treatment have significantly increased the use of oral therapies, and there are a number of new chemotherapeutic and biological drugs that are generally more convenient for health care institutions and patients, most of whom are treated at home. This has led to a major shift from directly observed, intermittent intravenous therapy to self-administered oral treatment, and raised the problem of adherence and safety. This is important in the case of oral anti-cancer drugs, whose poor tolerability and limited dosing options mean that they need to be actively monitored in order to avoid any serious complications or toxicities, unnecessary hospital visits or admissions, and unnecessary treatment reductions or interruptions, and maintain treatment activity [23-29].

The safety of home treatment has traditionally been handled by measures such as frequent medical visits, information leaflets, patient-held diaries, and phone contacts between clinicians/nurses and patients [30]. The key aspects of these processes are information and communication between patients and health professionals, but patient empowerment also plays a central role in the daily self-administration and management of oral therapies.

Telephone follow-ups for purposes of monitoring and providing health care advice have been widely used for many years but tend to be non-specific and time consuming [26,27,31]; however, mobile computing and communication technologies are beginning to play an increasing role in health care. There are a very few cases in which mobile phone messaging has been found to be beneficial in supporting the self-management of chronic diseases [32], but more advanced mobile phone systems that allow patients to alert health care professionals automatically in real time and only when necessary have been successfully piloted in the case of diabetes [33] and asthma [34].

The introduction of new-generation smartphones with computer-like features has made it possible to monitor of a whole series of behaviors using a wide range of sophisticated mobile-health (mHealth) apps designed to be used by health care professionals, patients, and even healthy people [5,35,36]. However, there has been a clear focus on chronic diseases (63%, primarily diabetes) and only 5% relate to cancer, as pointed in a recent review, although these have so far had little impact on public health outcomes [37]. A number of studies of the mobile monitoring of cancer patients have been published, including one randomized clinical trial, and the results have shown it can be effective, may reduce chemotherapy- or radiotherapy-related toxicity [38-41], and can even help to maintain maximum dose intensity in patients treated with oral capecitabine [42]. The patients involved in all of these studies generally felt reassured to be monitored at home, and the health professionals found that the system helped in the management of symptoms and promotion of timely interventions.

It has been argued that the contribution of eHealth technologies and mHealth apps to creating a more efficient and safer health care process can be maximized in a highly computerized setting [5,21,37]. This is the case in the province of Trento in northern Italy, where the regional health authority has introduced various eHealth solutions over the past 15 years that cover all public health activities, and are characterized by a high degree of integration and interoperability. They are not only routinely used to manage patients and support citizen and patient empowerment, but have also provided an opportunity for the development of new health care applications.

The aim of this paper is to describe two of these applications: the Safe Therapy Mobile (STM) system for the safe delivery of infusion chemotherapy in hospital wards, and the Onco-TreC home monitoring system, which has been designed to increase patient/health professional interactions in such a way as to improve the self-care capabilities and treatment adherence of cancer patients receiving oral therapies at home, and reduce or prevent the occurrence of toxicity and complications.

## Methods

### Information Technology Systems

The backbone of health technology in Trento is its hospital information system (SIO), which handles all of the patients’ clinical and administrative data, and is used by all of the public health care professionals working in the province. It can be accessed from every public health care facility, and supports various functions and activities including digital agendas and the paperless prescription of tests and drugs. General practitioners and primary care pediatricians are connected to the SIO by means of a virtual private network (VPN), which allows them to issue paperless drug prescriptions and receive all of their patients’ clinical data directly on their electronic desks.

A citizen-controlled clinical record system called the TreC (“three C”) system after its Italian name (*Cartella Clinica del Cittadino*) has been more recently introduced and integrated with the SIO, with the aim of empowering all citizens to manage their own health and facilitating communications with health care professionals and institutions [43]. The rationale underlying it is to provide a “safe place” in which to store personal health information and allow access to health-related public services such as their medical reports or monitoring services for chronic patients. The platform has two layers: “basic TreC services”, which consists of data management and other common Web-based functions, the most important of which is the authentication and authorization of users in order to ensure the security, integrity, and privacy of sensitive personal data, and “composite TreC services”, which includes higher integrated functions such as a structured health diary and monitoring tools for specific pathologies. Both layers interact with other mHealth solutions (“TreC access services and applications”) in order to allow users to take full advantage of them. The TreC platform is increasingly used and, as of 30 September 2014, more than 37,000 citizens had accessed more than 400,000 reports.

Finally, as long ago as July 2000, a Web-based, user-centered electronic Oncological Patient Record (eOPR) system (OncoSys) was developed in order to facilitate the clinical, organizational, and administrative management of all oncological patients in the region. It is integrated with the SIO and routinely used by our Medical Oncology and Radiotherapy units and six oncological day hospitals, and so far managed more than 27,000 oncological patients (for a total of 359,600 individual accesses). The characteristics and functions of the eOPRs (particularly the management of therapeutic regimens) have been previously described [44].

### The STM System

**Figure 1:**
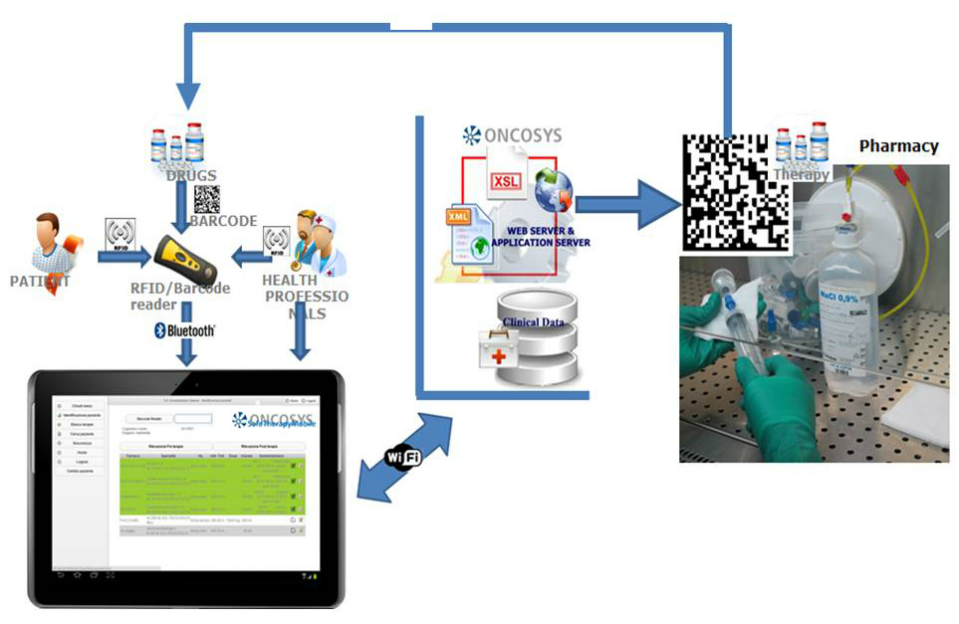
The basic components of the STM system. The eOPR originates a CPOE that is univocally associated with the patient’s RFID bracelet and the barcodes of the individual chemotherapy and ancillary drug, and washing solution. The RFID/barcode communicates via Bluetooth with the tablet, which communicates via Wi-Fi with the server of the OPR. The RFID/barcode reader checks the patient’s RFID bracelet, the nurse’s RFID, and the barcode on the infusion bag before each drug administration.

The STM system is a new application of our eOPR that has been designed and developed to support and monitor the entire process of drug medication in the hospital, from prescription to administration and reporting. Its design was preceded by modelling the workflow of patient therapy using Business Process Modelling Notation (BPMN), version 2.0 [45], and analyzing different tracking systems for mobile platforms and devices. It has a Web-based, multi-tier architecture: at the *business layer,* server and client interact to process the data in the *data layer*, which is visible to the user in the *presentation layer.* The system is cookie-free and no sensitive data can be intercepted because they remain on the server or are encrypted. One of its basic components is the eOPR, which includes a library of all the chemotherapy regimens currently being used, which have been reviewed by a group of experienced oncologists and pharmacists and electronically uploaded by informatic researchers, and support CPOE. The other components of the system are a radio frequency identification (RFID)/barcode reader, bar-coded drug labels, disposable RFID bracelets for patients, RFID tags for nurses, and a mobile device such as a tablet. The tablet communicates via Bluetooth with the RFID/barcode reader and via Wi-Fi with the server of the eOPR, in order to import the CPOE and export the eMAR, which contains the tracking data (Figure 1). When a chemotherapy regimen is prescribed, the eOPR originates a CPOE that is uniquely associated with the RFID bracelet of the specific patient. The CPOE details every single chemotherapy and ancillary drug (and the washing solution) in terms of dose, dilution volume, sequence, and infusion rate, which is also automatically associated with a specific barcode. The CPOE is sent via Web to the pharmacy for evaluation and drug preparation, and via Wi-Fi to the tablet. At the patient’s bedside, the RFID/barcode reader checks the patient’s RFID bracelet, the nurse’s RFID, and the barcode on the infusion bag before each drug administration in order to verify that the right drugs are administered to the right patient in the right sequence. In the case of an error (eg, wrong drug, wrong sequence, etc), the system blocks the procedure and prompts the nurse to correct it. The system tracks every drug administration: which nurse has administered which drug to which patient, the duration of each infusion, and the total duration of therapy. All of this information is entered in the eMAR and automatically recorded in the patient’s eOPR (becoming part of his/her oncological history), and may be used for clinical and/or organizational analyses.

The STM system was first repeatedly laboratory tested and then, in February 2014, was introduced into a day hospital with limited daily activities. At the beginning, the previous usual administration procedure and the STM system were used together in the same few volunteer patients but, after a few minor technical adjustments, the STM system was used alone for a total of 176 administrations. At the end of the testing period, it was adopted for routine chemotherapy administration in the initial day hospital and the more active day hospital of the Medical Oncology Unit of Trento.

The usability of the system and its acceptance by the nurses involved in the administration process was investigated using a modified questionnaire based on the “health IT usability evaluation scale” [46], which explored the three dimensions of the quality of working life, and the perceived usefulness and ease of use of the system (the fourth dimension of user control was not explored because the system had been designed in collaboration with the department and its introduction was preceded by extensive training of the nurses). The questionnaire was administered to all 15 nurses in both day hospitals after each had used the STM system for at least 2 months. An oral informed consent was obtained from nurses, whose participation was entirely voluntary.

### The Home Monitoring System

The home monitoring system was developed in order to deliver mHealth services in various medical contexts, and so relatively few technical enhancements are necessary to allow the same core components to be used for different clinical purposes and to support the different aspects of patient/doctor relationships. Based on the TreC platform, the architecture of the monitoring service is common to all chronic diseases, but the mobile phone or tablet user interfaces and parameters are specific for each condition.

**Figure 2:**
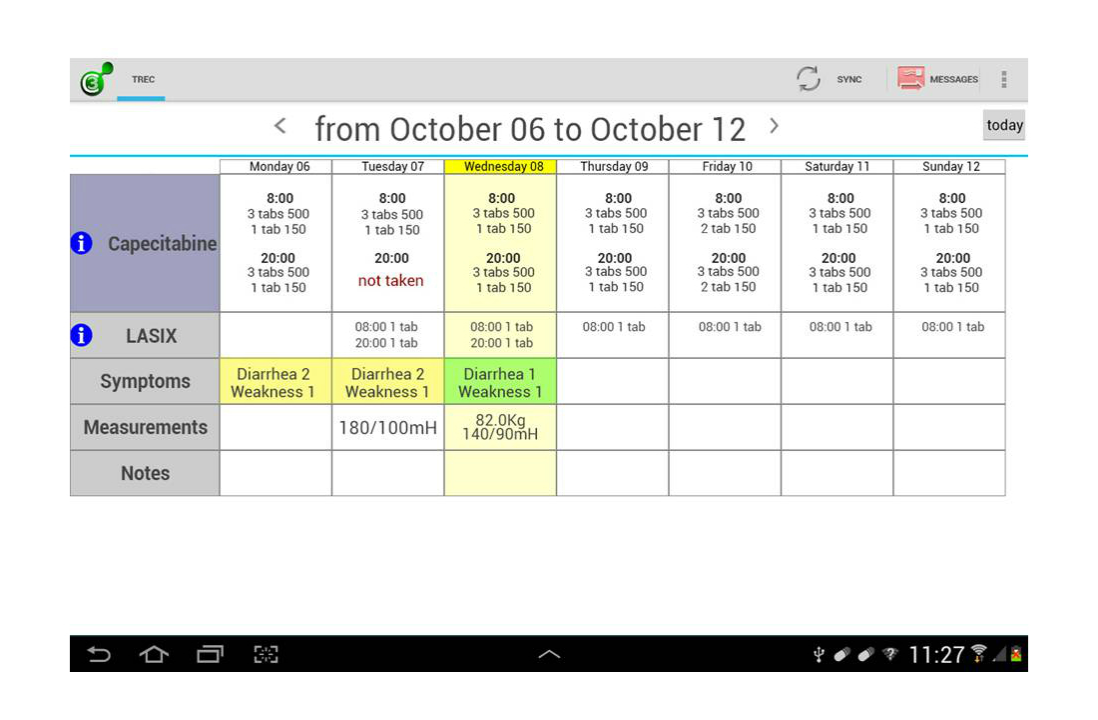
Screenshot of the mobile oncological diary showing patient’s prescribed therapy, self-assessed symptoms, and general data.

The system consists of a mobile diary and the Web dashboard. The mobile diary is an Android app that allows patients to record parameters related to their health (eg, blood pressure, weight, fever, specific disease symptoms, or therapy-related side effects) and the medications they have taken (see Figure 2); it also has a built-in rule-based alarm module that notifies health care professionals of critical conditions via email. All of the data are stored in a central database and made available in real time by means of the Web dashboard or a tablet. The Web dashboard allows health care professionals to check their patients’ data any time, and to monitor adherence to prescriptions and possible side effects. If a patient’s condition is a cause for alarm, he or she can be promptly contacted by a doctor or nurse.

The TreC home monitoring system has been tested in three pilot studies that used a living lab approach in real-life settings [47] and involved patients with chronic type I or II diabetes, hypertension, or youth asthma. The three studies were conducted on the basis of a similar 3-step evaluation process: (1) technical testing with a few (2-3) users, (2) qualitative evaluations based on a small sample of 10-12 patients, and (3) a validation clinical trial. The qualitative evaluations were made before and after the studies and consisted of audio-recorded, semi-structured interviews that were analyzed by means of template analysis [48] in order to evaluate the patients’ and clinicians’ perception of acceptability and usefulness.

The system has since been customized to meet the home management and remote monitoring needs of cancer patients treated with cytotoxic capecitabine or the biological agent sunitinib. Both drugs are widely used in clinical practice on a sufficiently long-term basis, and frequently require dose adjustments or support interventions in order to ensure patient safety and compliance, and maintain treatment activity.

This Onco-TreC system consists of the mobile diary app and Web dashboard, based on the TreC platform, which are closely integrated with the eOPR, which originates the CPOE and records the administered therapy and related events. The mobile oncological diary app is deployed on a tablet to be used by the patient, and contains sections relating to the prescribed drugs, symptoms, general data, and day-by-day notes.

**Figure 3:**
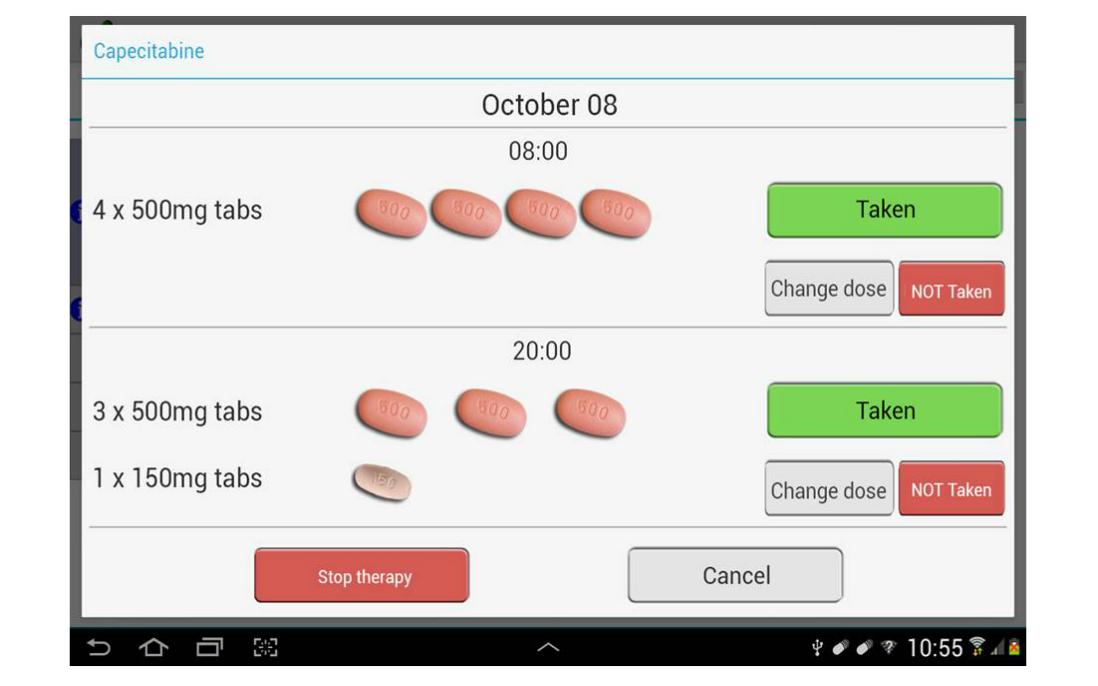
Screenshot of pills and buttons.

In the drug section, the CPOE is automatically converted to the number and type of pills that the patient has to take each day throughout the duration of the treatment. The patients are required to enter data into the system manually at least once a day by clicking on specific buttons each time they take the drug or not for any reason (Figure 3).

**Figure 4:**
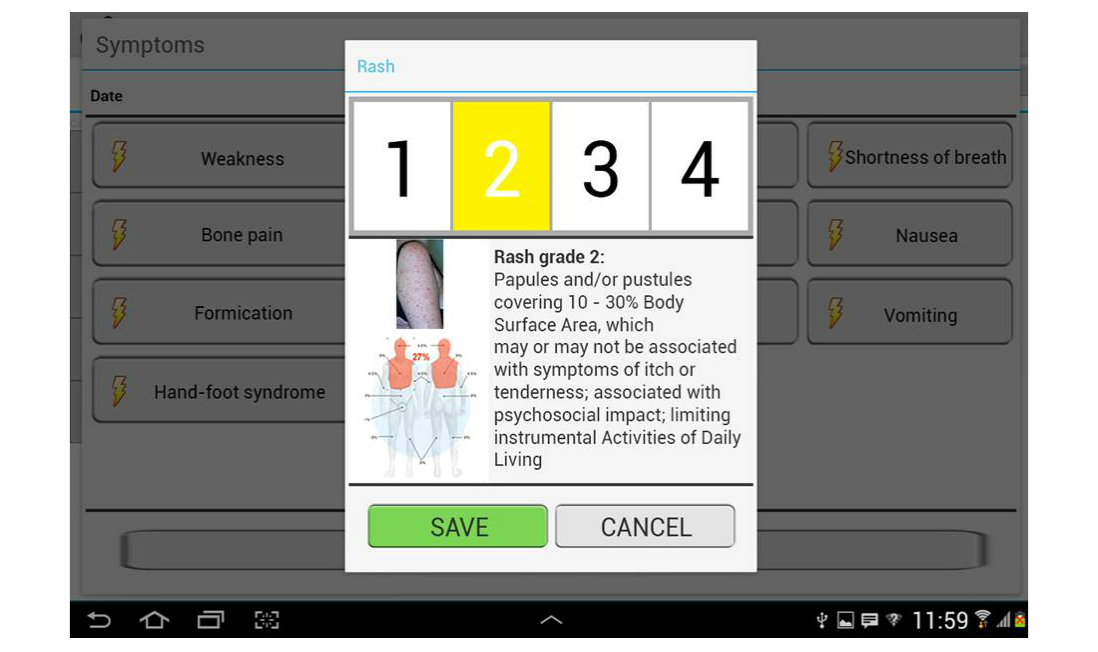
Screenshot of mobile oncological diary showing the window that allows patients to input onset and intensity of therapy-related rash. The app helps patients determine grade of toxicity by displaying explanatory texts and pictures.

In the symptoms section, patients can choose from a number of predefined, drug-specific side effects. Adverse events are graded and summarized on the basis of the NCI-CTCAE, Version 4.02 [49], which is available in the app: the patients are asked to indicate the grade with the help of a scale defined in simple language and, in the case of skin toxicities, illustrated by pictures (Figure 4).

Every time such data is entered, the patient is given suggestions for action (eg, stop/continue the therapy or follow instructions), a feature that integrates and reinforces the patient information provided during a preliminary education phase [50]. All of the toxicity data, together with general data such as blood pressure, weight, fever, and patient notes, appear in the patient’s diary and on the Web dashboard, and are recorded in the patient’s eOPR.

**Figure 5:**
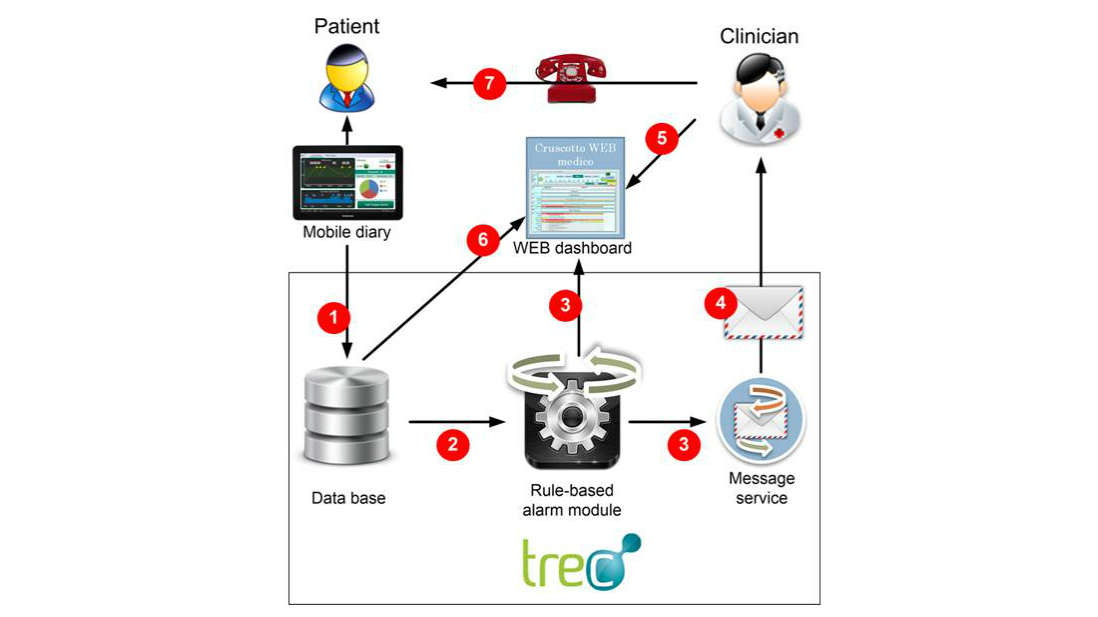
Conceptual model of cancer patient home monitoring: (1) diary compilation - data are stored in central database and displayed on dashboard; (2) real- time analysis by rule-based alarm module; (3) If “critical event” is detected, alarm signal is automatically generated and displayed on dashboard; (4) message service alerts competent health professional; (5) doctor/nurse accesses patient dashboard to evaluate patient’s problems; (6) dashboard shows patient’s data and alarms; (7) they can contact patient directly if necessary.

The alarm module has also been customized using oncological drug-specific rules, which generally define any grade 3 toxicity symptom as an alarm signal that is automatically notified by email to the health professional responsible for monitoring the patient and displayed on the dashboard (Figure 5).

**Figure 6:**
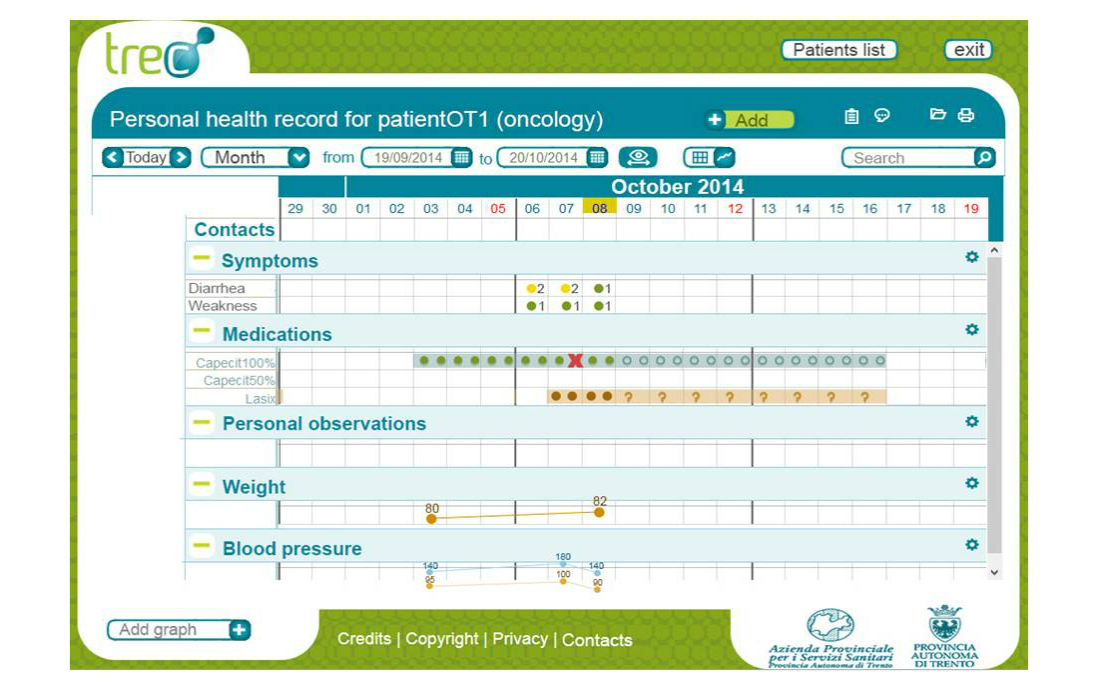
The oncological dashboard via Web browser. The horizontally tiled charts show general data, eg, weight, fever, symptoms, personal observations, and drugs (taken or discontinued).

The Web-based dashboard (Figure 6) consists of a set of horizontally tiled time-based charts that show the programmed therapy and the set of monitored data entered by the patients via their mobile diaries, thus allowing oncologists and nurses to check the patients undergoing treatment at a glance, in a defined time window (eg, 3 days, 1-3 weeks, 1 month), assess any problems, and provide appropriate and timely indications. The nurses are organized on a rotating basis in order to ensure the 24-hour coverage of alarms.

The development and lab testing of Onco-TreC have now been completed, and the system will soon be validated by means of a prospective study of 60 consecutive patients designed to verify adherence to therapy, the prevention of home complications, dose reductions, or treatment interruptions, and any unscheduled access to a day hospital or emergency room, and assess its usability and acceptance by patients and health care professionals. The evaluation will be made using a customized version of the “health IT usability evaluation scale” [46] in order to investigate the four dimensions of the quality of working life (for health care professionals), communication (for patients), perceived usefulness and ease of use, and user control. We will also investigate the patients’ perceived quality of life using the Functional Assessment of Cancer Therapy-General (FACT-G) questionnaire [51], and anxiety levels using the Hospital Anxiety and Depression Scale (HADS) questionnaire [52], both of which will be administered in a training phase at baseline, and after 6 and 12 weeks of treatment.

The following results therefore refer to the testing and validation of the original systems.

## Results

### The STM System

By the end of the testing period, the system had been used to administer a total of 176 treatments to 59 patients. Only nine of the treatments were not completed during the first 2 weeks because of minor technical reasons such as a few short periods of weak Wi-Fi network signals and some difficult to read drug label barcodes, all of which were easily resolved. The system never failed to match the patient/drug/nurse combination correctly (and recognized errors when we voluntarily tried to change a drug or sequence), and proved to be accurate in tracking the time and duration of any single drug administration, the name of administering nurse, and the duration of the entire treatment. In order to test impact of the system on hospital workflows (the duration of the visits, therapies, and waiting times of each patient), we evaluated the duration of the entire administration process in a few patients who received the same chemotherapy regimen before and after the system was introduced into routine practice: there were no significant differences in the duration of chemotherapy administration, although this was not the perception of the majority of the nurses.

Analysis of the questionnaires showed that the users were generally satisfied with the use of STM (13/15, 87%; Q.8) and positively perceived all of the considered dimensions (the quality of working life, and the perceived usefulness and ease of use of the system) (Table 1).

For the purposes of this paper, the most interesting data concerning the perceived usefulness of the system when managing therapy administration (87% (13/15); Q.7); the improvement in information sharing (93% (14/15); Q.6); and the general perception of greater safety when administering the therapies (87% (13/15); Q.11) as the system is perceived as helping to associate the prescribed drugs with the right patient (73% (11/15); Q.9), and respect the correct sequence of administration (93% (14/15); Q.10).

It is more difficult to evaluate the nurses’ perception of the system’s impact on the speed of executing nursing tasks: 60% (9/15), said that it slowed down operations at the patients’ bedsides (Q.4), but 80% (12/15) said that it speeds up recording the details of the administered therapies in the eOPR (Q.5). Before the introduction of the system, the nurses wrote the time they started the infusion of each drug on a paper form and, at the end of their shift, manually entered the data in the OPR, whereas the STM system relieves them of these tasks by automatically recording the data and entering them in the eOPR (thus also eliminating possible transcription errors).

**Table 1:** Results of the nurses’ administered questionnaire on the STM (Safe Therapy Mobile) system (n=15).

			Strongly disagree	Disagree	Agree	Strongly agree
			n (%)	n (%)	n (%)	n (%)
**Quality of working life**						
	1	STM has improved a nurse’s work	1 (7)	1 (7)	12 (80)	1 (7)
	2	STM has improved the work of our ward	1 (7)	2 (13)	11 (73)	1 (7)
	3	STM is important in treatment management	0 (0)	2 (13)	6 (40)	7 (47)
**Perceived usefulness**						
	4	Using STM quickens the management of therapies at the patient’s beside	4 (27)	5 (33)	6 (40)	0 (0)
	5	Using STM quickens the recording of therapy details in the OPR	0 (0)	3 (20)	6 (40)	6 (40)
	6	Using STM improves the sharing of information about the administration process	1 (7)	0 (0)	10 (7)	4 (27)
	7	STM is useful when managing the administration of therapies	0 (0)	2 (13)	5 (33)	8 (53)
	8	I am generally satisfied with STM	1 (7)	1 (7)	12 (80)	1 (7)
	9	STM makes it more difficult to make a mistake in associating therapy and patient	0 (0)	4 (27)	3 (20)	8 (53)
	10	STM makes it more difficult to make a mistake in the sequence of the administered drugs	0 (0)	1 (7)	5 (33)	9 (60)
	11	Using STM makes me feel safer when administering the therapies	2 (13)	0 (0)	5 (33)	8 (53)
**Perceived ease of use**						
	12	Learning to use STM was easy	0 (0)	3 (20)	9 (60)	3 (20)
	13	STM is easy to use	0 (0)	2 (13)	10 (67)	3 (20)
	14	I can always remember how to use STM	0 (0)	0 (0)	12 (80)	3 (20)

### Home Monitoring of Chronic Patients

A total of 43 patients were involved in the three pilot studies: 20 with type I or type II diabetes, 15 with hypertension, and eight with youth asthma. These pathologies were chosen in order to include different mixes of patient self-care and empowerment and the direct intervention of doctors and nurses: type I diabetes and youth asthma are mainly managed autonomously by patients and their families, whereas type II diabetes and hypertension require health care professionals to play a more active role in monitoring and evaluating data.

Some of the results were common to all studies, whereas others show that the mobile remote monitoring app has different effects depending on the duration of the study and the distribution of the workload between doctors and patients*.*

All of the studies found that the system was well accepted by patients for up to 3 months [53-55], and the health care professionals were positive toward the system because of its novelty (type 1 diabetes [56]), its potential for reducing unnecessary face-to-face encounters (type 2 diabetes: submitted), and its diagnostic reliability (hypertension [53]).

However, the patients involved in two studies perceived the system as intruding on their everyday lives and causing an additional burden [53,55]. This tension between potential benefits and perceived intrusiveness is well described by a simulation based on the real data of patients with type I diabetes, a chronic disease which is usually managed by the patients themselves. During the 6 months of the test, the system alerted doctors 95 times and, each time, the doctors were asked what they would have done had the system been implemented in clinical practice. In 14% of cases (13/95), they would have contacted the patient (or his/her parents) straight away and, in 58% of cases (55/95), they would have closely monitored the patient’s data; in the remaining 28% of cases (27/95), they would have simply waited for the next visit. These data were presented to the patients at a project meeting, and it became clear that patients thought they could manage by themselves the condition that had triggered the alarm to doctors, and that they considered the system as a means of supporting self-management rather than remote monitoring. This led to a request that doctors intervene only on call [51] and a request to redesign the alarm module to receive notifications on any conditions of attention and limiting to a small fraction the number of alarms automatically sent to health professionals.

With regard to the conditions characterized by a greater need for remote monitoring, the patients appreciated the closer medical supervision, and considered the system a useful means of reducing the need for direct contacts with health professionals and increasing their perception of safety [53]. This situation can be considered very similar to that of oncological patients treated with oral anticancer therapies at home.

The preliminary results of these pilot studies show that clinicians and patients perceive the approach as useful and reliable, but it is also necessary to pay attention to the expectations that mHealth solutions may raise in users [53-56].

## Discussion

### The STM System

The published data clearly suggest that the integration of EMR, CPOE, and BCMA systems can decrease medication errors and help to deliver safer and more efficient care. This makes it highly suitable in oncology because specifically designed, integrated, and interoperable systems, together with good patient/health professional communications and robust Web or Wi-Fi connectivity, are vital components ensuring the safety of the administration of chemotherapy [5,6,7].

Bearing these principles in mind, our STM system is fully integrated with the eOPR that we routinely use for the total management of all oncological patients, and so any chemotherapy treatment can be entirely managed from prescription (automatically transformed into a CPOE for the pharmacy) to administration at the patient’s bedside, where each single drug is checked by the barcode reader in order to verify that the right drug is administered to the right patient in the right sequence. The system has proved to be accurate, reliable, and capable of guaranteeing the safety, monitoring, tracking, and recording of the entire treatment for each patient, and has been successfully used for the last 5 months at a busy day hospital for adult oncological patients.

Even the best health technology is designed not to replace health care professionals, but to enhance their ability to care for their patients, and so it is always important to consider its impact on the workflow of health care providers and the way in which it is perceived. In addition to verifying its ability to guarantee the safe administration of chemotherapy, the STM has been evaluated in terms of its usability and acceptability in a department staffed by a quite stable group of specialized and experienced nurses who are able to ensure a high standard of care. These nurses have found that it supports their work in at least three ways. First of all, its use removes a potential source of clerical errors by digitalizing and automatically transferring information from the barcode/RFID scanner to a tablet and then the eOPR, thus replacing the previous paper-based system. Second, the system monitors and tracks the entire infusion process, and all of the information is transferred to the eMAR and automatically recorded in the patient’s eOPR to became a part of his/her oncological history; this means that all of the nurses are aware of every stage in the administration process in real time, thus increasing the sharing of information. Third, the questionnaire data suggest that the introduction of the technology is perceived by nurses as improving the quality of their work and professional skills.

Our nurses had a positive perception of all of the dimensions considered in the questionnaire (the quality of their working life, and the usefulness and ease of use of the system), except for the fact that the system appeared to slow down bedside operations. This observation is not new and probably reflects the impact of the new technology on the workflow of health professionals, who are generally reassured as soon as they become more familiar with the technology and more efficient at using the system [7]. This view is supported by the fact no significant objective differences in the duration of chemotherapy administration were found after the system was introduced.

In conclusion, it seems that our STM system can simplify the medication process by eliminating some unnecessary steps, and that its safety features not only make cancer treatments safer for patients, but improve the accuracy and efficiency of the process of ordering, preparing, and administering antineoplastic drugs for health care workers.

### Home Monitoring

The use of mobile health apps is not new in the field of home monitoring of chronically ill and oncologic patients as well [37,42,57-60]. In this context, mHealth seems to be a particularly attractive means of managing conditions that require patients to be monitored or cared for at home because, given the widespread use of mobile connectivity, it can enhance information sharing with clinicians as a result of real-time communications. The greatest perceived benefits of the more widespread adoption of mHealth solutions included improvements in health care system processes, the collection and retrieval of crucial medical data, and the ability of patients to manage chronic conditions [61]. Specifically, some of these studies have shown that mobile monitoring of cancer patients can be effective, may reduce chemotherapy- or radiotherapy-related toxicity [38-41], and can even help to maintain maximum dose intensity in patients treated with oral capecitabine [42]. Although eHealth technologies and mHealth apps have so far had little impact on public health outcomes [37], it has been argued that their contribution to creating a more efficient and safer health care process can be maximized in a highly computerized setting [5,21,37].

The architecture of the home monitoring system described in this paper is not new, but its use has been extended. It is based on the broad, multipurpose TreC platform, which was designed to deliver mHealth services in various medical contexts, which means that relatively few technical enhancements were necessary to allow the same core components to be used in the setting of oncology. The minimum set of basic components (mobile phone or tablet apps) to be used by patients have been previously evaluated in three different patient populations involving different mixes of patient empowerment and self-care, and different interventions by doctors and nurses [53-55]. All of these studies showed that the system was well accepted by patients and considered useful by physicians, although they also showed that attention needs to be paid to the expectations that mHealth solutions may raise in users as the remote monitoring led to different effects depending on the duration of the study and the distribution of the workload between doctors and patients [53].

Nevertheless, the results were sufficient to provide a rationale for developing the Onco-TreC system for monitoring patients treated at home with oral capecitabine and sunitinib. Such patients are traditionally given appropriate education and information, and are always asked to call health staff in the case of problems. However, this obviously excludes off-duty hours, holidays and nights, and, together with the difficulties that may occur when communicating with a hospital, may give rise to feelings of abandonment and, in some cases, the need to seek access to the Emergency Department.

However, what makes Onco-TreC quite different from other apps is the fact that it is integrated in a system that has been specifically developed for the total management of cancer patients. The patient-held diary combines patient-reported symptoms with the real-time detection and communication of potentially serious adverse events [28], and gives doctors better information concerning toxicity and compliance to therapy, thus allowing prompt intervention and supporting patient adherence. All of the information automatically becomes part of each patient’s clinical history and is immediately available whenever any decision-making support is needed.

Moreover, this highly integrated, complex, and composite information system guarantees privacy, security, interoperability, and (particularly) connectivity, thus real-time patient/health professional communication. All of these features, together with the automatic alarm system should have a beneficial impact on the quality and efficiency of health care. Our home monitoring app certainly still has to be validated before it can be considered helpful in clinical practice, but this will soon be done in a prospective study of patients treated with oral oncological drugs.

In conclusion, our approach to designing and implementing an integrated oncology management system using mobile apps was aimed at ensuring the safer in-hospital delivery of infusion chemotherapy and empowering cancer patients to manage their disease and treatment at home.

Mobile apps such as STM and Onco-TreC may play a role in creating an organizational culture of safety, but it needs to be remembered that, even when human processes are replaced by integrated, computerized activities in order to increase safety, a major element remains the importance of staff training and patient education and empowerment.

## Abbreviations

ASHP: American Society of Health-System Pharmacists
BCMA: barcode-assisted medication administration
BPMN: business process modelling notation
CDSS: clinical decision support systems
CPOE: computerized prescriber order entry
eMARs: electronic medication administration records
eOPR: electronic oncological patient record
EMR: electronic medical record
EPRs: electronic patient records
IOM: institute of medicine
ITs: information technologies
mHealth: mobile Health
RFID: radio frequency identification
SIO: hospital information system
STM: safe therapy mobile
